# Supplementary material for: Content-rich biological network constructed by mining PubMed abstracts
Source: BMC Bioinformatics. 2004 Oct 8;5:147. doi: 10.1186/1471-2105-5-147 (PMC528731; doi:10.1186/1471-2105-5-147)
Supplement: Additional File 2 — The original results of the above study (non-essential files are deleted to keep the file size under the limit set by BMC bioinformatics). [file 1471-2105-5-147-S2.bz2 › chilibotAdditionalFile2/dip10/379ID9927421E9792/html/NGFR_NTRK3.html]

 


 **NGFR** and **NTRK3** 
  
Found 76 abstracts in PubMed, retrieved 10.  
 

 What does Google say? 
 PDF only 
| .edu only 

---

**Interactive relationship** (e.g. stimulation, inhibition, etc)

**Non-interactive relationship** (e.g. studied together, co-existance, homology, etc.)

- Mouse PTPRO mRNA was observed in neuron populations expressing TrkA, the high affinity nerve growth factor receptor  [ **NGFR** ] , or TrkC  [ **NTRK3** ] , the neurotrophin 3 receptor  [ **NTRK3** ] , and immunoreactive mPTPRO and TrkC  [ **NTRK3** ]  colocalized in large dorsal root ganglia proprioceptive neurons.  Ref: 12532410 J Comp Neurol, 2003
- Our results show that myelin formation is inhibited in the absence of functional p75NTR  [ **NGFR** ]  and enhanced by blocking TrkC  [ **NTRK3** ]  activity.  Ref: 12424382 Science, 2002
- Thus p75NTR  [ **NGFR** ]  and TrkC  [ **NTRK3** ]  receptors have opposite effects on myelination.  Ref: 12424382 Science, 2002
- ... the expression of the neurotrophin, BDNF, the NT 3 receptor, TrkC  [ **NTRK3** ] , and the nonselective, low affinity neurotrophin receptor p75NTR  [ **NGFR** ] , were comparable to wild type.  Ref: 11848682 Neurobiol Dis, 2002
- Neurotrophin signaling is mediated by Trk family tyrosine kinases such as TrkA, TrkB and TrkC  [ **NTRK3** ] , as well as by the pan neurotrophin receptor p75NTR  [ **NGFR** ] .  Ref: 11836532 Nat Neurosci, 2002
- Although neurotrophins did not regulate p75NTR  [ **NGFR** ]  processing, the alpha and gamma secretase mediated cleavage of p75 is modulated by receptor tyrosine kinases Trks TrkA and TrkB but not TrkC  [ **NTRK3** ] .  Ref: 12843241 J Neurosci, 2003
- Sections of pancreatic cancer and normal pancreatic tissue were examined by immunohistochemistry for the expression of the neurotrophins NGF, BDNF, NT 3, NT 4, and their receptors TrkA, TrkB, and TrkC  [ **NTRK3** ] , as well as the low affinity receptor, p75NTR  [ **NGFR** ] .  Ref: 11561004 J Histochem Cytochem, 2001
- We have used immunofluorescence techniques with a panel of antibodies against known neurotrophin receptors trk A, trk B, trk C  [ **NTRK3** ] , p75NTR  [ **NGFR** ]  to map the locations of these receptors in the developing neuromuscular system of mice from our neurotrophin 3 NT 3 knockout colony.  Ref: 12141445 Int J Dev Biol, 2002
